# Implementation of targeted next-generation sequencing for the diagnosis of drug-resistant tuberculosis in low-resource settings: a programmatic model, challenges, and initial outcomes

**DOI:** 10.3389/fpubh.2023.1204064

**Published:** 2023-08-03

**Authors:** Leonardo de Araujo, Andrea Maurizio Cabibbe, Lusia Mhuulu, Nunurai Ruswa, Viola Dreyer, Azaria Diergaardt, Gunar Günther, Mareli Claassens, Christiane Gerlach, Christian Utpatel, Daniela Maria Cirillo, Emmanuel Nepolo, Stefan Niemann

**Affiliations:** ^1^Molecular and Experimental Mycobacteriology Group, Research Center Borstel, Leibniz Lung Center, Borstel, Germany; ^2^Emerging Bacterial Pathogens Unit, IRCCS San Raffaele Scientific Institute, Milan, Italy; ^3^Department of Human, Biological & Translational Sciences, School of Medicine, University of Namibia, Windhoek, Namibia; ^4^National TB and Leprosy Programme, Ministry of Health and Social Services, Windhoek, Namibia; ^5^Department of Pulmonology and Allergology, Inselspital, Bern University Hospital, University of Bern, Bern, Switzerland

**Keywords:** Next generation sequencing (NGS), *Mycobacterium tuberculosis* complex (MTBC), NGS clinical use, Genomic diagnostic, Genomic surveillance, NGS programmatic implementation, high TB burden countries, low- and middle-income countries

## Abstract

Targeted next-generation sequencing (tNGS) from clinical specimens has the potential to become a comprehensive tool for routine drug-resistance (DR) prediction of *Mycobacterium tuberculosis* complex strains (MTBC), the causative agent of tuberculosis (TB). However, TB mainly affects low- and middle-income countries, in which the implementation of new technologies have specific needs and challenges. We propose a model for programmatic implementation of tNGS in settings with no or low previous sequencing capacity/experience. We highlight the major challenges and considerations for a successful implementation. This model has been applied to build NGS capacity in Namibia, an upper middle-income country located in Southern Africa and suffering from a high-burden of TB and TB-HIV, and we describe herein the outcomes of this process.

## 1. Introduction

Infectious diseases are currently one of the most explored fields for clinical and public health genomics, as sequencing technologies simplified and accelerated the deep characterization of pathogens (1). Pathogen genomics is transforming surveillance programs allowing both prompt identification of outbreaks and epidemics and accurate diagnosis at individual level, replacing the standard techniques in microbiology laboratories (2, 3). The emergence of infectious threats, such as SARS-CoV-2 and Monkeypox viruses, showed the needs of strengthening health systems worldwide with implementation of NGS capacity (4). However, other more prevalent diseases such as tuberculosis (TB) and malaria should also profit on NGS implementation to improve diagnosis and for monitoring/surveillance.

TB is a leading infectious killer, after COVID-19 in 2020/2021, with estimated total incidence of 10.6 million new cases and 1.6 million deaths (5). It is also the leading killer of people living with human immunodeficiency virus (HIV) and a major contributor to deaths related to antimicrobial resistance. The use of World Health Organization (WHO)-recommended molecular diagnostics (mWRDs) for diagnosis and drug resistance (DR) testing to at least key drugs such as rifampicin, isoniazid and fluoroquinolones, remains limited in low-resource, high TB burden settings. The incomplete drug sensitivity testing (DST) coverage leads to empiric treatment initiation, despite the existing treatment guidelines requiring access to testing (6).

Whilst mWRDs already accessible in low- and middle- income countries (LMICs) allow prompt resistance prediction for one or few drugs, next generation sequencing (NGS) of *Mycobacterium tuberculosis* complex (MTBC) strains offers the most comprehensive approaches to determine resistance to the current recommended regimens (3, 6). Two main NGS-based approaches may be used: whole genome sequencing (WGS) and targeted NGS (tNGS). tNGS takes advantage of the selective amplification of DR-related regions of MTBC genome and provides quick results directly from clinical specimen, with higher sensitivity than WGS, lower turnaround time and easier interpretation (7–10).

Genome sequencing has also already been introduced as a tool to investigate TB DR evolution, transmission dynamics and the population structure of MTBC, for surveillance of DR (9–11), and patient’s management (12), although a roadmap to a programmatic implementation of TB genomics is still lacking.

Recent investigations have shown that using sequencing to inform treatment regimens for DR TB led to decisions comparable to those derived from phenotypic DST (pDST) (6, 7). Also, it became evident that analyzing by NGS all genes known to be associated with DR would improve the design of personalized multidrug-resistant (MDR) TB regimens (high concordance with pDST-informed decisions) (13). However, the implementation of NGS in TB clinical laboratories requires adequate infrastructure, training, and strategic planning. Challenges include procurement, sample referral, quality-assured procedures, data management, translation into clinical practice and sustainability (e.g., human resource retention) (14, 15). Therefore, it is important to collect data-driven evidence from practical implementations in high TB burden countries. Many of the challenges to an effective implementation of genomics in resource-limited settings are technical and deal with the renovation of healthcare systems, including high costs, suboptimal supply, inadequate infrastructure and link of sequencing information to existing record systems (1, 16, 17). Other aspects involve social and ethical components (use/sharing of data and clinical application thereof).

Herein we detail our model of implementation of tNGS for DR prediction of MTBC strains in settings with no or low previous sequencing capacity. We detail how this model was implemented at the University of Namibia (UNAM) in Windhoek, Namibia, one of the 30 high-burden countries for TB and TB-HIV.

## 2. Implementation model

In this section we define the steps that we consider crucial and how those were addressed during the tNGS implementation in Namibia (*in italics*).

### 2.1. Implementation strategy and roadmap

**Figure 1:**
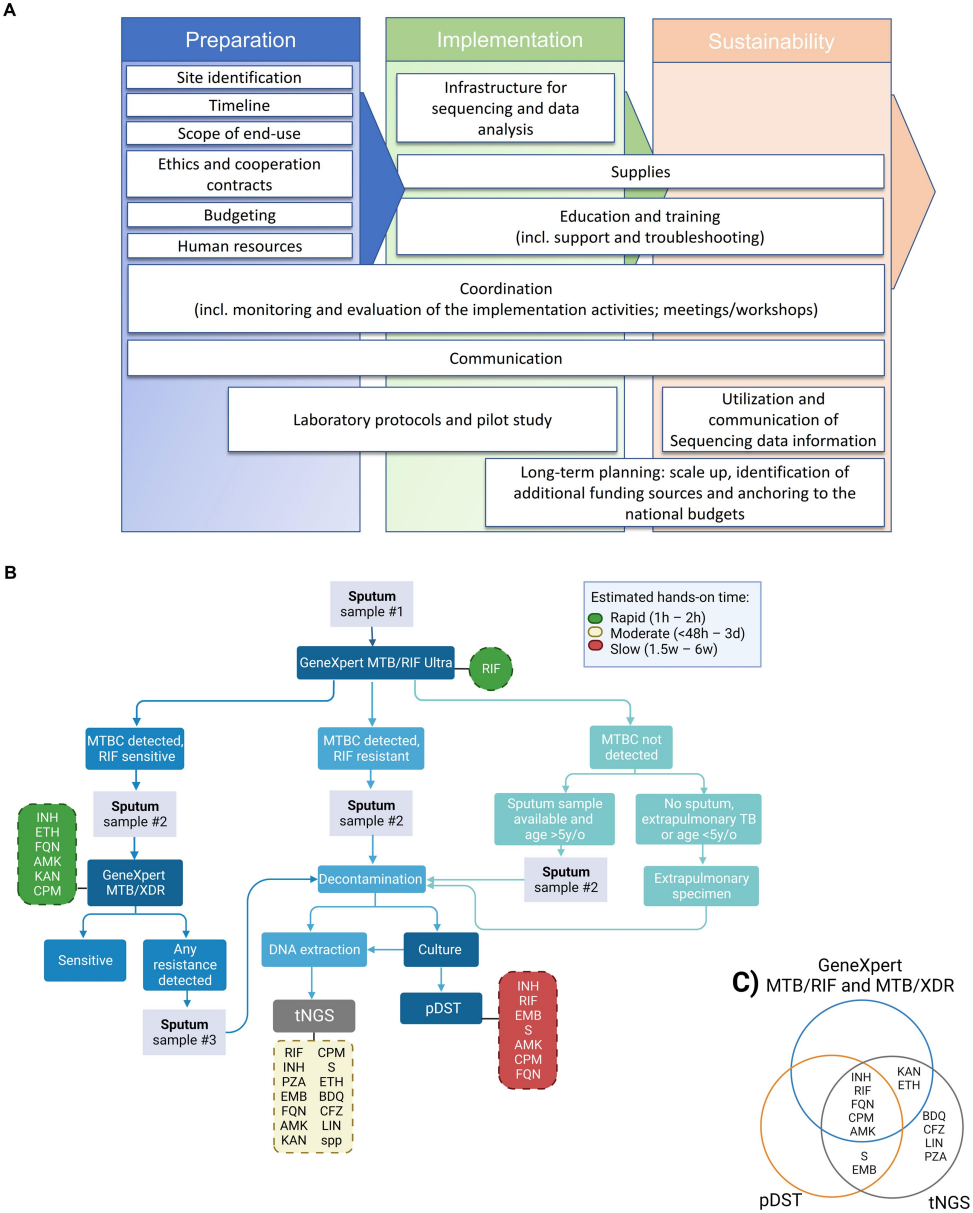
Proposed tNGS implementation roadmap, diagnostic flowchart and tested drugs for the detection of drug-resistance TB cases including NGS technologies for enhanced genotypic resistance prediction of *M. tuberculosis* complex (MTBC) strains. **(A)** Roadmap for building tNGS capacity in low- and middle-income countries (from left to right). **(B)** Flowchart including the GeneXpert MTB/RIF Ultra and MTB/XDR (or any other endorsed test with similar application) as screening tests for selection of samples to downstream targeted next-generation sequencing (tNGS). Hands-on time refers specifically to the theoretical amount of time needed to perform the test, excluding the time needed to collect the clinical specimen. Turn-around time (TAT) refers to the theoretical amount of time needed to perform the test added to the theoretical time to have available clinical specimens (specially affected when additional samples have to be collected). The TAT for tNGS in routine settings is expected to be around 6–8 days from the entry test result. Dashed boxes indicate the panel of drug-resistances investigated by each test, green, yellow and red colors indicate the relative hands-on time, reference: (12). **(C)** Venn diagram depicting the panel of drug-resistances investigated by each test, worth mentioning that the number of amplified targets is different between the molecular DST tests. pDST, phenotypic drug sensitivity testing; INH, isoniazid; RIF, rifampicin; FQN, Fluoroquinolones; CPM, capreomycin; AMK, amikacin; S, streptomycin; EMB, ethambutol; KAN, kanamycin; ETH, ethionamide; BDQ, bedaquiline; CFZ, clofazimine; LIN, linezolid; PZA, pyrazinamide.

As shown in Figure 1A, our strategy for the implementation of NGS was based on three pillar phases: preparation, implementation, and sustainability. These phases are subdivided into smaller categories of tasks (Figure 1A, white boxes).

In the preparation phase, as the first phase of the implementation process, we defined the main outcomes of the intervention and the strategy to assess deliverables. The entire outline of the implementation process must be planned here.

The implementation phase focuses on practical work once the strategies have been developed. Capacity building, support, training, pilots and the search for sustainability begin in this phase.

The sustainability phase aims to scale up NGS capacity, anchor NGS in local guidelines and help programs in the search for new sources of funding.

Our implementation strategy was based on the exchange of knowledge between a center of expertise for NGS (non-profitable), in this case with extensive experience in doing NGS on clinical MTBC strains, to another center in a LMIC that does not had this capacity.

#### 2.1.1. Preparation phase

##### 2.1.1.1. Site identification

Epidemiological context, laboratory network and testing algorithms are considered in our approach. In this context, we needed to consider the local testing algorithm for TB and DR TB, in order to determine the best way to incorporate tNGS. We needed to identify which specimens could be used to extract mycobacterial DNA (e.g., leftovers of sputum specimens, new sputum, cultures, etc.) by avoiding unnecessary additional steps to the standard procedures for collecting and preparing specimens.

*Namibia has an estimated TB incidence of 460/100,000 population and estimated 560 multidrug-resistant (MDR) TB cases per year. Xpert MTB/RIF Ultra and line probe assays (LPA) 1st-2nd line are used. The diagnostic algorithm is reported in* Supplementary Text*. Second line DST testing coverage is incomplete due to reagent stock outs or culture contamination leading to shipment of selected strains for further testing outside the country. The NGS platform was implemented at an academic institution, the UNAM, within the national TB programme (NTP) network. Consultative need assessment meetings were held between the UNAM, the National TB and Leprosy Programme and collaborating stakeholders (Research Center Borstel and Robert Koch Institute, Germany, Ministry of Health and Social Services and the Namibian Institute of Pathology) which facilitated and supported the implementation of tNGS in Namibia. Additionally, in Namibia the use of tNGS for TB diagnosis is supported by the NTP as one recommendation of the end term review of the NTP is “to develop a contingency plan and diversify TB testing, particularly drug resistance testing.”*

##### 2.1.1.2. Timeline

The complete timeline must be realistic and focused on priorities to mark the overall project progress.

*Our NGS implementation process in Namibia started in 2019 with a 1-year long preparation phase, during this period, among other tasks, the protocols were developed and tested at the center of expertise for NGS. After that, the capacity building started, i.e., the implementation phase, with an initial aim to be done in 2-years. However, our timeline was severely affected by COVID-19 pandemic, the implementation phase was duly extended having a total duration of 4 years (2019–2022)*.

##### 2.1.1.3. Scope of end-use

The overarching goal of the implementation process is to develop local technical capacity for the use of tNGS as an add-on diagnostic tool for prediction of DR profiles of medicines included in MDR or rifampicin-resistant (RR) TB regimens. A proposed workflow for the future incorporation of tNGS into the national algorithm is shown on Figure 1B. Our proposed algorithm would include samples for tNGS that presented prior DR profile on screening test(s), i.e., GeneXpert MTB/RIF Ultra and MTB/XDR. tNGS would enable faster results than culture-based pDST, with the additional benefit of interrogating genotypic resistance to bedaquiline, clofazimine, linezolid and pyrazinamide (currently not tested in the local settings, Figure 1C).

*Initially, around 50 MDR/RR TB samples are expected per year mainly from the TB referral hospital in Windhoek and will be used for pilot adoption of tNGS, then it is planned to roll out to cover MDR/RR-TB cases from the entire country*.

##### 2.1.1.4. Ethics and cooperation contracts

Ethical review ensures that the study adheres to the agreed ethical standards and in this field with the specific consideration of sharing of genetic data and personal data.

*The project in Namibia was approved by the local Ethics committee of UNAM and approved by the Ministry of Health (# 17/3/3EN). Cooperation and material transfer agreements were also signed by the implementing partners and the implementation site, containing the scope of the process and terms for collaboration*.

##### 2.1.1.5. Budgeting

A financial plan must include costs for key actors and activities including personnel, infrastructure, purchase of devices and consumables, shipments, training (including travel and accommodation), internet and other services.

*In* Supplementary Figure 1 *we reported a rough average percentage of expenses per year over the 4 years of implementation in Namibia. Human resources (HR) at the referral center and implementation sites are the highest expense (52%), followed by consumables (23%), and devices (17%). The total funds used in this period was approximately 418,500.00 USD. With regards to equipment maintenance, as we acquired new equipment, it came with the standard manufacturer’s warranty for at least the initial implementation phase. After that, extended warranty plans were considered for just the sequencing devices and were contracted with the official local distributors of the sequencers. The other equipment was included in the regular institutional maintenance activities, being under UNAM responsibility*.

##### 2.1.1.6. HR

A survey of available laboratory staff must be carried out to understand if reallocation of existing staff is feasible. If new staff is hired, training needs should be considered. The calculation of needed personnel was done empirically based on our experience as center of expertise for NGS, available funds, and expected sample flow.

*A laboratory technologist and a PhD student were recruited to be dedicated to the development of tNGS activities locally, based on their background in medical laboratory sciences and medical biochemistry, with molecular biology techniques experience such as Sanger sequencing*.

##### 2.1.1.7. Coordination

Defining the coordination process is an essential part, coordinators supervise all processes (HR, materials and equipment) and ensure that all team members are aware of the objectives, schedule and progress. Resources must be allocated efficiently, potential risks anticipated and mitigation strategies applied.

*Implementation coordinators were hired/delegated at the international center of expertise for NGS and locally*.

##### 2.1.1.8. Communication

Effective communication of results is a key step to motivate all stakeholders (Ministry of Health (MoH), staff and the community).

*Annual workshops were carried to share the implementation outcomes with main stakeholders. Concurrently the tNGS was discussed regularly with NTP and clinical partners in order to secure an early translation of implementation into clinical practice (ongoing), based on the tNGS data currently being generated in-country during implementation*.

#### 2.1.2. Implementation phase

##### 2.1.2.1. Infrastructure for sequencing and data analysis

This capacity involves the selection of the adequate facilities for the installation of the NGS lab. A wet lab infrastructure herein refers to the physical laboratory space, equipment, and reagents required for the pre-PCR area (DNA extraction), and the post-PCR area (PCR amplification, library preparation, and sequencing). The sequencing laboratory should have adequate space, sturdy benchtops, electricity and internet outlets, and follow strict room temperature, humidity, and air quality requirements for operation of the sequencers and related instruments.

*The sequencing apparatus was installed in the lab of the department of Human, Biological and Translational Medical Sciences in collaboration with the “Group Research in Infectious Diseases (GRID), UNAM (*Supplementary Figure 2*). Two Illumina iSeq100 machines were purchased as an upgrade to the existing Sanger sequencing facility. The iSeq100 instrument was selected based on its size, multiplexing capacity suitable for local workflows, cost-effectiveness, and user-friendly interface. The user’s manual was referred to find the appropriate site for installation*.

A dry lab infrastructure refers to the computational infrastructure required for analyzing and interpreting the sequencing data generated by tNGS.

*The sequencers were connected to the network, to a network attached storage system for data backup and to an uninterrupted power supply device. Fridge and freezers for the reagent’s storage and a thermal cycler were also procured. Due to power interruption experienced at the implementation site a backup freezer was installed with backup power supply. Computers were purchased for routine use, which are sufficient for analyzing tNGS in the cloud. Additionally, a high-end computer was purchased to handle more complex local analysis in case further needs are identified (*Supplementary Table 1*)*.

##### 2.1.2.2. Supplies

An efficient and trustworthy supply chain is crucial for achieving sustainability.

*Materials were securely delivered either from the Research Center Borstel, Germany, and shipped, or through regional distributors (however mainly located in South Africa)*.

*We performed a rough estimation of the initial investment needed to procure the consumables required to start the tNGS activities (*Supplementary Table 2*)*.

##### 2.1.2.3. Education, training, and support

After the training and competency assessment, continued assistance should be conducted (using instant messaging apps, regular meetings, and written reporting methods).

*In Namibia, personnel were trained on tNGS workflow and analysis, including hands-on, quality checks, and run of the iSeq100 instrument. Training was performed either hybrid or locally at the implementing facility. The educational activities comprehensively included theoretical and practical packages with tailored agenda, refresh, and troubleshooting sessions over the entire implementation phase. Data analysis requested additional tutoring activities and was not limited to the laboratory study staff*.

##### 2.1.2.4. Laboratory protocols and pilot study

Upon completion of the practical sequencing training, local samples from TB patients are sequenced as pilot to assess the capacity and feasibility.

*Strains from clinical culture TB samples were subjected to tNGS as pilot (No. 48 RR as identified by Xpert MTB/RIF Ultra). The details and outcomes of this study are described in* Supplementary Text *and* Supplementary Table 3.

## 3. Discussion

NGS-based analysis of clinical MTBC strains bridge gaps associated with pDST and the limitations of other mWRDs for DR testing (12). tNGS can detect DR-associated mutations to a variety of antibiotics as those included in the WHO-recommended DR TB treatments, and is applied to a variety of clinical specimens (9, 10, 18). It also allows species identification at the lineage level, detection of mixed populations and heteroresistance (9, 10, 18).

It is expected that within the next few years, with an increased automation of the NGS workflow and improved treatment algorithms, NGS workflows will be widely implemented at least at reference laboratory level and clinicians will adopt individualized treatment decisions soon after diagnosis of DR TB is made by entry tests from patient samples (ideally 6–8 days in programmatic conditions), adjusting also the duration of treatments, and therefore their efficacy, costs and toxicity (6).

The process of implementation of TB genomics for surveillance of DR TB, recommended by the WHO, and as a routine diagnostic tool at country level, requires careful planning, strong commitment, and investment to support successful adoption into national algorithms. Before the implementation process starts, it is crucial to ponder the use of NGS within the NTP, with implications for choice of technologies and equipment to use, selection of sites, referral systems, target turnaround times, implementation of clinical decision making and incorporation into treatment guidelines. An implementation plan is needed to build the NGS infrastructures (wet and dry) and HR (management and technical). In addition to a careful planning step, it is also important to proceed stepwise and to report the obstacles identified during the implementation process. If the implementation strategy is not adequate, based on evidence, and without regular assessments, this tool will not positively impact the diagnosis at implementation sites. In fact, considering the several barriers to NGS uptake at country level, a completely ‘self-sustainable’ NGS capacity seems still far from reality in LMICs (15). Reassuringly, laboratory infrastructures and specialized academic education for scientists and clinicians are expanding quickly in LMICs, opening new opportunities for research in such scenarios, further progress, awareness and equitable partnerships (19, 20). With the COVID-19 pandemic, the number of countries recognized by the African Union having local access to sequencing facilities increased by around 50% and consortia were created to study the spread of SARS-CoV-2 variants (21). Worth mentioning that the NGS capacity developed within the project in Namibia was timely used for emergency response to COVID-19 pandemic and variant’s surveillance. In this context and given costs, countries should consider that an investment for a given disease (e.g., surveillance of DR TB or COVID-19) can impact other health priorities such as viral diseases or AMR surveillance, as it creates facilities and capacity that can be expanded. Another unforeseen benefit was that the trained personnel at UNAM were able to train other laboratory scientists from the Sub-Saharan region (Botswana, and Eswatini).

Some challenges to a programmatic implementation of NGS are technical and include the complexity of protocols and workflows, and sophisticated and not yet fully standardized data management, analysis and interpretation. To tackle this, several collaborative initiatives were created in order to provide data on the performance of existing technologies, and industries and researchers are developing end-to-end user-friendly NGS solutions. Furthermore, knowledge of molecular mechanisms at the basis of the emergence of DR is incomplete, limiting the predictive values of NGS. The correct use of data requires training of clinicians on their interpretation (Figure 1A, utilization and communication of sequencing data information), a competence that in initiated but still faces challenges in Namibia. Herein, the analysis of our pilot data indicates that the protocols need to be properly validated at the implementation sites, and that the limitations of tNGS have to be evaluated locally. We had to start the implementation with DNA from MTBC cultures for ease, although the immediate next step is to apply it on primary patient samples (e.g., sputum) with protocols already validated. On the brighter side, the analysis of the pilot samples showed a clear advantage of tNGS by providing a broader vision of resistance profile to anti-TB drugs (Figure 1C), the user-friendly analysis interface, validation steps and the indication of the “usability” of the sequencing results.

The main challenges of the NGS implementation process at UNAM were: (i) initial technical issues experienced with the iSeq100 setup, later resolved through remote technical support; (ii) unforeseen costs to stabilize and control the room temperature required due to the local semi-arid environment; (iii) an unstable internet connection has resulted in a challenging data upload process; (iv) delay in the construction of dedicated pre-PCR and post-PCR areas; (v) delay in the delivery of material due to COVID-19 restrictions; and (vi) sufficient adhesion of local stakeholders to translate tNGS results into clinical practice and public health policy. Despite these challenges, our planning and initial implementation phase are finalized, NGS capacity was successfully built and is currently in use by the GRID, UNAM, while the implementation of tNGS results into clinical processes, and some other competencies of the sustainability phase, have just started. We recognize the need for a clinical advisory committee (CAC), that will review and discuss the reports generated by tNGS, as well as provide guidance to the clinicians. The CAC should consist of representatives from NTP and National TB Reference Laboratories, implementing partners, laboratory specialists, clinicians, and international experts. However, the implementation of such committees and the approval to use tNGS data requires multisectoral and political support in the country. This process is ongoing and challenging, but it should be facilitated upon the release of WHO guidelines for tNGS use in DR TB diagnostics. The plan is that the tNGS DR report and standard results will be shared with the CAC, which will review and discuss the data and provide guidance to the clinicians.

Logistic aspects, such as procurement and supply chains in countries where distributors are not present and unable to provide optimal maintenance and support, importation requirements, as well as transportation of samples or reagents in case of unreliable referral systems, represent threats in the current scenario. In Namibia, the obstacles primarily revolve around the market and logistic, there are complete shortages of some products in the local market. As a solution, we have managed to identify suppliers of sequencing products in neighboring countries, particularly South Africa. However, these are only third-part distributors, and the materials are imported from other countries. Consequently, there is a significant loss of shelf-life during transportation between manufacturer-distributor- implementation site; increased prices are also expected due to the reselling process. As a feasible alternative, but not long-term sustainable, the products can be purchased at the center of expertise for NGS outside the country (in a place where the supplier availability, market prices and logistics are less challenging), re-packed and sent to the implementation site. Another important, yet often unforeseen obstacle that greatly affects the availability of NGS supplies, is the bureaucratic and time-consuming processes for importation of donated goods. This still requires facilitation by the local authorities. WHO and other TB stakeholders are encouraging companies involved in the manufacturing/supply of NGS-related items to explore ways to expand the use of genomics in LMICs and make it more accessible, through incentives such as modified pricing models, reduction of costs and loans at low-interest (22). The development of reagents/technologies that do not require temperature control can also help to push the implementation of NGS in Sub-Saharan Africa.

The SWOT (strengths, weaknesses, opportunities, and threats) analysis reported in Table 1 offers a framework to assess the characteristics of the TB genomics solution and helps to identify the main areas for improvement, and strategies to maximize the advantages and to mitigate the disadvantages of tNGS in TB control and care. We proposed here a model based on tNGS implementation, which is more suitable than WGS for direct and faster DST testing, as it usually does not require culture. This approach offers higher standardization of wet protocols as kit-based, automated data analysis pipeline, and increased multiplexing compared to WGS for routine settings. Conversely, WGS approach from cultures would provide higher resolution of genomes and transmission outbreaks, but currently with a more challenging implementation process in high TB burden, low-resource settings.

**Table 1:** SWOT analysis for use of tNGS-based genomics in TB control and care.

**Strengths**	**Weaknesses**
❖ Multi-purpose, multi-disease	❖ Need of genotypic-phenotypic associations
❖ Suitable from various sample types	❖ Turnaround time depends on sample referral and sequencing capacity/multiplexing
❖ Rapid (faster turnaround times than conventional pDST testing)	❖ Start-up costs
❖ Kit-based and user-friendly analysis tools (improved standardization)	❖ Currently not feasible at peripheral level
❖ Deep level of genetic information enabling “precision”	❖ Procurement and supply chain
	❖ Need of specialized and trained personnel
**Opportunities**	**Threats**
❖ Less phenotyping in routine testing	❖ Borderline mutations
❖ High predictive value for drug-resistance	❖ Confidence-grading of mutations requires large and representative datasets
❖ Huge research on innovative NGS technologies	❖ Support to clinicians
❖ Development of lists of confidence-graded mutations reflecting on routine Nucleic Acid Amplification Tests	❖ Not all resistance mechanisms can be explored (e.g., gene expression, structural changes)
❖ Interrogates resistance to additional anti-microbials not routinely tested in national algorithms	❖ Information technology (IT) infrastructure
❖ Research outcomes	❖ Cost-effectiveness to be demonstrated
	❖ Efficient and timely results reporting
	❖ To achieve sustainability

This table provides a Strengths, Weaknesses, Opportunities, and Threats (SWOT) analysis of the use of genomics in TB control and care. The SWOT analysis helps to identify strategies to maximize the advantages and mitigate the disadvantages of having tNGS capacity for TB implemented.

The ultimate scope of the tNGS implementation in routine settings of high TB and/or DR TB incidence countries is to improve clinical management of cases and provide surveillance to resistance to new regimens. Relevant TB stakeholders are looking with interest at the pilot implementation studies and findings and should encourage roll out at the level of national/regional laboratories (23). It is advisable that, once tNGS receives approval from WHO as a diagnostic tool for DR TB, NTPs will include tNGS in the diagnostic algorithms. This should be accompanied by sustainability plans and budgetary allocations. In settings where NGS capacity is lacking, the implementation process can be better planned by leveraging the experience presented in this study.

Sustainability can be achieved by several measures, starting with the release of WHO policies on NGS use for clinical care/surveillance, then with the inclusion in the Global Drug Facility list for regular global supply at negotiated price, the adoption of NGS into national guidelines, and the development of more cost-effective protocols and other commercial point-of-care solutions. Furthermore, the incorporation of sequencing as valuable public health tool into the national anti-TB DR surveys will facilitate the collection of comprehensive data from countries. This data will inform timely public health actions to be integrated into national strategic plans for TB. It will enable the design of optimized diagnostic algorithms, assessment of the efficacy of recommended treatment regimens, identification of research needs, and guide resource allocation planning (24).

## Data availability statement

The datasets presented in this study can be found in online repositories. The names of the repository/repositories and accession number(s) can be found at: https://www.ebi.ac.uk/ena/, PRJEB62858.

## Author contributions

LA, AC, LM, NR, VD, GG, CG, DC, EN, and SN conceived the idea and designed the study. LA, AC, LM, VD, CU, EN, and SN analyzed and interpreted the data. LM, AD, VD, and CU generated the sequencing data. LA, LM, AD, GG, MC, CG, EN, and SN coordinated the implementation. LA, LM, AC, GG, and NR designed the proposed diagnostic workflows. LA, AC, and LM wrote the initial draft of the manuscript. DC, EN, and SN supervised the study. All authors contributed to obtaining and assembling the data, during the review process, data interpretation, critical review of the manuscript, and approved the final version of the manuscript.

## Funding

Implementation of NGS in the country was supported by the German Global Health Protection Program – Federal Ministry of Health, the SeqMDRTB_NET (ZMVI1-2519GHP708, https://ghpp.de/en/projects/seqmdrtb-net/).

## Conflict of interest

The authors declare that the research was conducted in the absence of any commercial or financial relationships that could be construed as a potential conflict of interest.

## Publisher’s note

All claims expressed in this article are solely those of the authors and do not necessarily represent those of their affiliated organizations, or those of the publisher, the editors and the reviewers. Any product that may be evaluated in this article, or claim that may be made by its manufacturer, is not guaranteed or endorsed by the publisher.

## Supplementary material

The Supplementary material for this article can be found online at: https://www.frontiersin.org/articles/10.3389/fpubh.2023.1204064/full#supplementary-material
