# Supplementary material for: Implementation of targeted next-generation sequencing for the diagnosis of drug-resistant tuberculosis in low-resource settings: a programmatic model, challenges, and initial outcomes
Source: Front Public Health. 2023 Aug 3;11:1204064. doi: 10.3389/fpubh.2023.1204064 (PMC10478709; doi:10.3389/fpubh.2023.1204064)
Supplement: Supplementary file 1 [file Data_Sheet_1.docx]

**Implementation of targeted next-generation sequencing for the diagnosis of drug-resistant tuberculosis in low-resource settings: a programmatic model, challenges, and initial outcomes.**

**Running title:** Implementation of NGS-based diagnosis for DR-TB in high burden countries

**Authors:** Leonardo de Araujo 1†, Andrea Maurizio Cabibbe 2†, Lusia Mhuulu 3†, Nunurai Ruswa 4, Viola Dreyer 1, Azaria Diergaardt 3, Gunar Günther 3,5, Mareli Claassens 3, Christiane Gerlach 1, Christian Utpatel 1, Daniela Maria Cirillo 2≠*, Emmanuel Nepolo 3≠, Stefan Niemann 1,3≠

† These authors contributed equally to this work and share first authorship

≠ These authors share last authorship

* corresponding author

Affiliations:

1 – Molecular and Experimental Mycobacteriology Group, Research Center Borstel, Leibniz Lung Center, Borstel, Germany

2 – Emerging Bacterial Pathogens Unit, IRCCS San Raffaele Scientific Institute, Milan, Italy

3 – Department of Human, Biological & Translational Sciences, School of Medicine, University of Namibia, Namibia

4 – National TB and Leprosy Programme, Ministry of Health and Social Services, Windhoek, Namibia

5 – Department of Pulmonology and Allergology, Inselspital, Bern University Hospital, University of Bern, Bern, Switzerland

Address correspondence to: Daniela M. Cirillo, [cirillo.daniela@hsr.it](mailto:cirillo.daniela@hsr.it)

# Supplementary data

**Reporting model –** For reporting of our implementation model and pilot study, we considered the Standards for Reporting Implementation Studies (StaRI) by describing the strategy behind the implementation process and the intervention performed [[26]](https://www.zotero.org/google-docs/?E59hMt). We defined the context for the intervention, expectations on its impact, success, adaptation, and implications.

**Standard algorithm** – Already established local diagnostic algorithms for detection of DR TB require collection of one sputum sample for an initial screening for rifampicin resistance using Xpert MTB/RIF Ultra, followed by a second sputum specimen that is submitted for further testing such as smear microscopy, LPA, pDST (Suppl. Figure 3). The country diagnostic algorithm suggests LPA testing for second line drugs for MDR/RR-TB and pDST for first line drugs (excluding pyrazinamide) and fluoroquinolones. All RR strains are referred to a centralized reference laboratory in the capital city for phenotypic and genotypic AST. Selected strains are tested for further drug-resistance characterization to new drugs at the laboratory at the National Institute of Communicable Diseases in Johannesburg (South Africa). The pilot study collected a total of 48 MTBC RR samples (based on GeneXpert MTB/RIF assay) upon informed consent from participants for tNGS after culture.

**Genomic DNA Extraction –** For validation of the technique, MTBC cultures were stored in BACTEC MGIT 960 at Namibia Institute of Pathology, National Reference Laboratory. Genomic DNA was extracted from culture positive samples using CTAB protocol as described previously [[27,28]](https://www.zotero.org/google-docs/?PlaJSf).

**tNGS –** Targeted NGS was performed using the commercially available assay Deeplex Myc-TB (Genoscreen, France) for prediction of resistance to 13 anti-TB drugs. The extracted DNA underwent 24-plexed Deeplex PCR according to the user manual. Libraries were generated from these amplicons using the Baym protocol, a modified and more cost-effective NexteraXT (Illumina) protocol [[25]](https://www.zotero.org/google-docs/?SVnl0Z) and loaded on an Illumina iSeq100.

**Data analysis –** After sequencing runs were completed, raw FASTQ files were uploaded to the Deeplex web application for automated data analysis. The analysis tool presents internal methods for assessing the sequencing quality. These methods include the validation of the positive control (PC, containing DNA of *M. bovis* BCG), the internal amplification control (non-mycobacterial DNA) and the negative control. Herein we observed the sequencing result acceptability, which is a grade to the overall capacity to detect mutations in resistance-associated positions varying from +++ (can identify mutations present in 3% to 100% of the sequencing reads), ++ (10% to 100%), + (80% to 100%), - ( ≥1 resistance-associated locus not covered by the sequencing reads); coverage breadth and average depth. After sequencing, controls are validated based on criteria of Deeplex Myc-TB manual (<https://deeplex.bluebee.com/deeplex/>).

**Pilot study –** In this phase, the laboratory procedures are validated at the sites and sequencing quality is assessed. During the Preparation phase the SOP were adapted and validated at the reference lab in Germany. Then, in Namibia, the pilot runs were performed between March 2021 and January 2022. In total 4 tNGS runs were performed with in-person support from external experts during the initial implementation period and yielded a successful result. Each run contained 3 controls and between 3 to 18 samples (more samples can be included per run, but we decided based on the availability of samples and the need of practice of the local staff). The completed runs passed the control validation step. The PC in these runs showed 100% composite target coverage breadth and an average coverage depth varying from 721.7x to 2920.8x, however yielding different sequencing acceptability results (Suppl. Table 3).

Among the 48 MTBC samples analyzed with Deeplex Myc-TB, it was observed a composite target coverage breadth ranging between 99.78% and 100%, and average coverage depth 630.7x to 1944.8x was obtained. The majority (31/48, 64.5%) of the sequenced samples showed a sequencing acceptability result of +, i.e. the quality of the sequencing data was enough to detect resistance-associated mutations with high prevalence among the reads. Only a single sample demonstrated negative (2%) sequencing result acceptability, 15 (31.3%) showed ++ and 2 (4.1%) showed the highest quality grading (+++). DR profiles were diverse and are reported in the following Suppl. Table 3.

# 2. Supplementary Figures


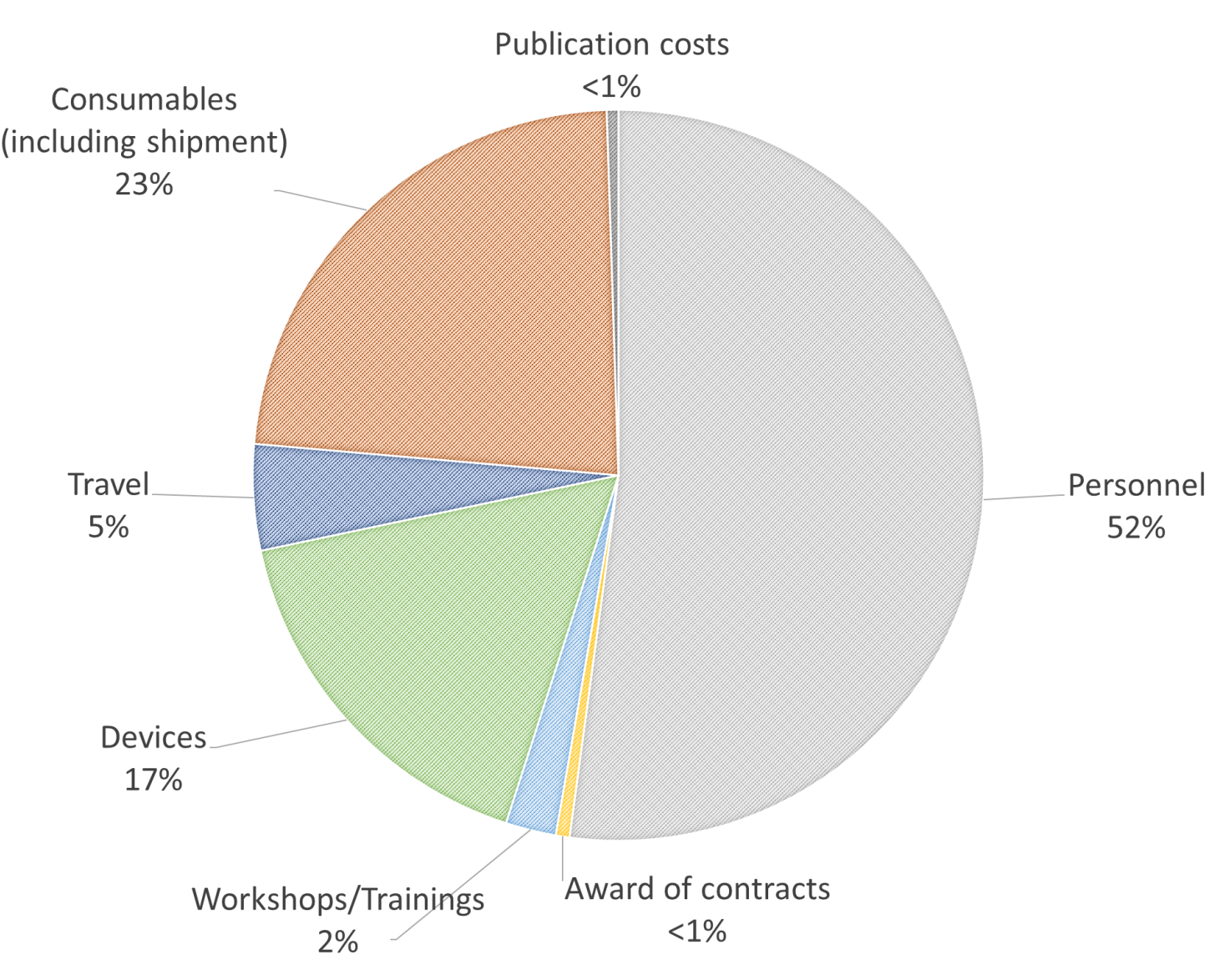


Supplementary Figure 1 - Average costs per category during four years of preparation and implementation in Namibia. The costs were calculated based on rough estimates and may vary depending on specific circumstances. The data presented here is intended to provide a general overview of the expenses associated with implementing a project of this nature. It should be noted that these figures are averages and individual costs may differ depending on factors such as project size, scope, and location. Overall, this figure serves as a useful reference for stakeholders involved in planning and budgeting for similar projects.


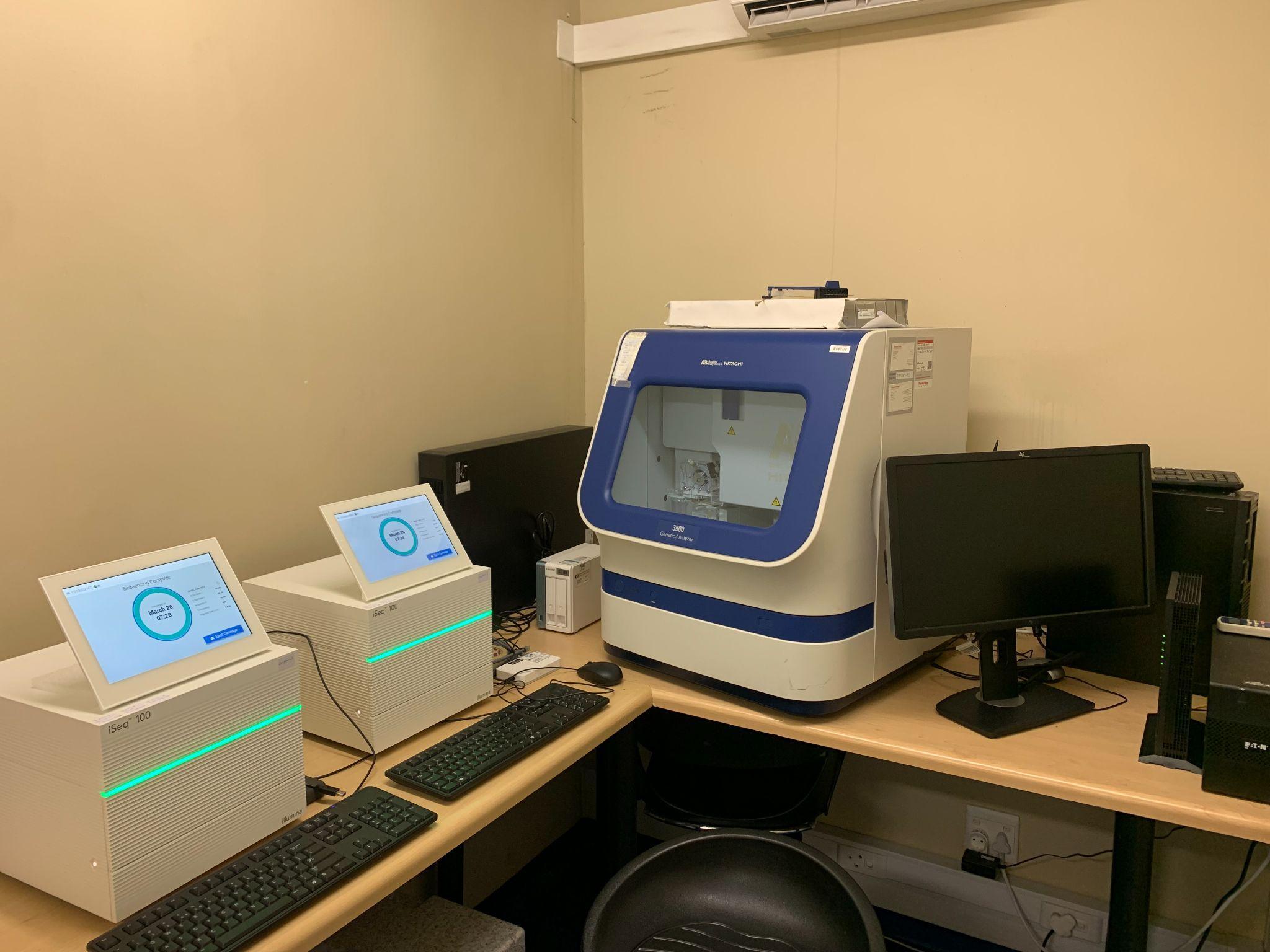


Supplementary Figure 2 - NGS lab infrastructure in the lab of the department of Human, Biological and Translational Medical Sciences in collaboration with the research group for Research in Infectious Diseases, University of Namibia, Windhoek, Namibia.

#


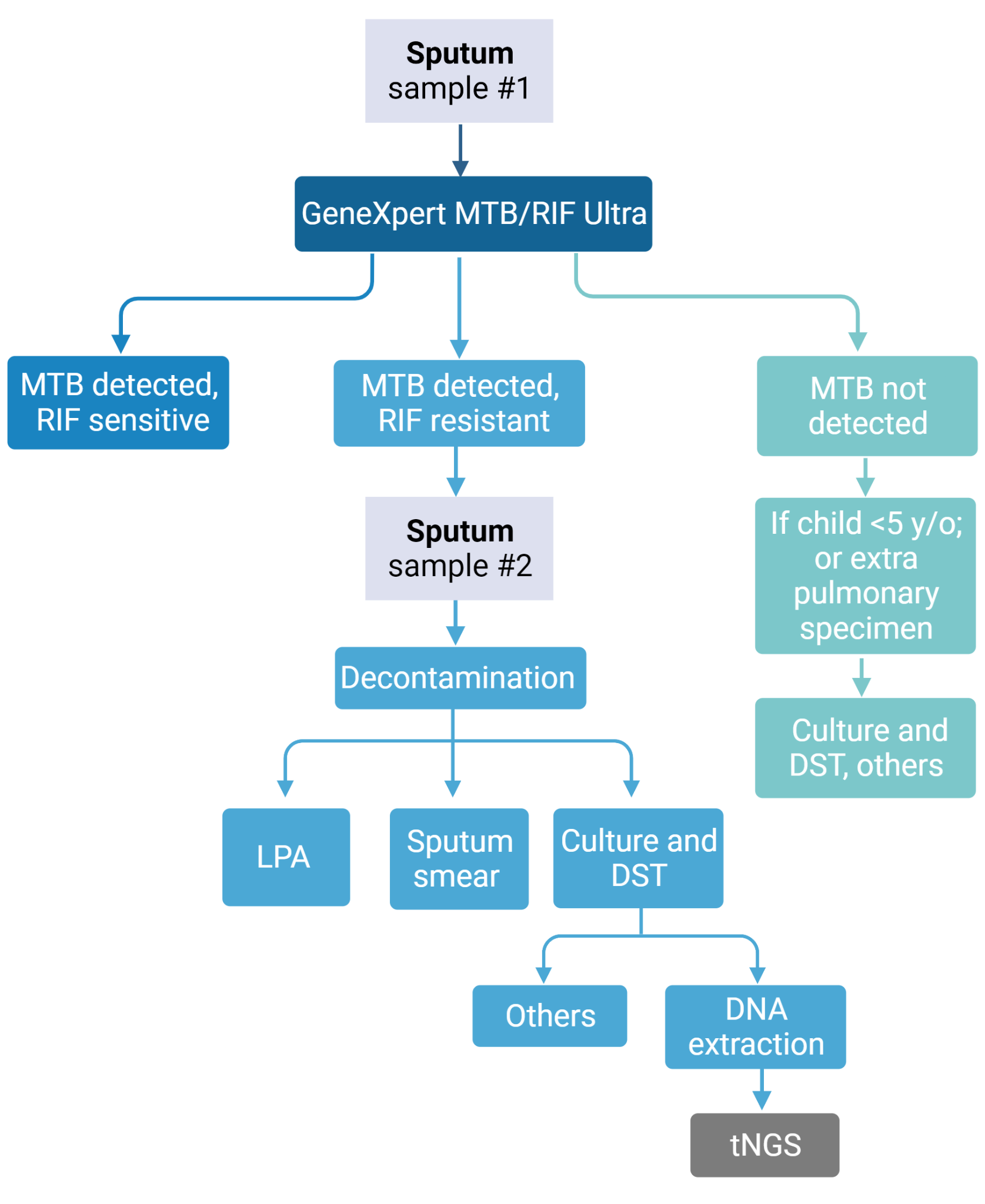


Supplementary Figure 3. Simplified sample enrollment flowchart used to detect DR TB cases, the additional branch was included for this study (in grey). tNGS was only used on samples screened as RR by GeneXpert MTB/RIF Ultra.

# Supplementary Tables

Supplementary Table 1. Specifications, quantity and costs of the equipment procured for building the local NGS infrastructure. The quantities listed in this table may vary depending on the specific needs of the site/project, and the costs are subject to change depending on several factors, such as vendor pricing and shipping costs. This table serves as a reference for stakeholders involved in the planning and procurement of equipment for NGS implementation projects.

| **Category (purpose)** | **Specifications** | **Manufacturer** | **Quantity installed** | **Price per unit (USD)** | **Sub-total (USD)** |  |
| --- | --- | --- | --- | --- | --- | --- |
| Computer (for routine use) | 15.6" FHD (1920X1080) | Dell | 2 | 815 | 1,630.00 |  |
|  | Intel core i5 |  |  |  |  |  |
|  | 8GB (1X8GB) 2666MHZ DDR4 |  |  |  |  |  |
|  | 256GB SSD |  |  |  |  |  |
|  | Windows 10 pro (64bit) |  |  |  |  |  |
| Computer (for local data analysis) | Intel core i7 | Dell | 1 | 2,727.50 | 2,727.50 |  |
|  | Nvidia quatro RTX 4000, 8GB |  |  |  |  |  |
|  | 32GB 2X16GB DDR4 2666MHZ |  |  |  |  |  |
|  | 2TB 5400RPM SATA 2.5" HDD |  |  |  |  |  |
|  | 512GB PCIE |  |  |  |  |  |
| PCR Workstation | PCR Workstation 32inch including air filters | AirClean | 1 | 6,222.00 | 6,222.00 |  |
|  |  |  |  |  |  |  |
| Thermal cycler | T100 | BioRad | 1 | 3,281.00 | 3,281.00 |  |
| Freezer | 254 liters | Defy | 1 | 437.00 | 437.00 |  |
|  | 481 liters | Defy | 1 | 820.00 | 820.00 |  |
| Fridge/Freezer | 208/115 Liters | Defy | 1 | 328.00 | 328.00 |  |
| Uninterruptible power supply device (for proper turn-off in case of blackouts) | Tescom 3 KVA UPS | Tescom | 2 | 553.8 | 1,107.60 |  |
| Network switch | D-Link Network Switch DGS-1008 | D-link | 1 | 30.31 | 30.31 |  |
|  |  |  |  |  |  |  |
| Network attached storage | QNAP 2-BAY Network attached storage DRIVE, 1.7GHZ | QNAP | 1 | 290 | 290.00 |  |
| Internal hard drive (for data back-up) | Seagate 2TB 3.5" HDD SATA | Seagate | 2 | 441 | 882.00 |  |
| Sequencer | iSeq100 | Illumina | 2 | 21940 | 43,880.00 |  |
|  |  |  |  |  |  |  |
| Total |  |  |  |  | 61,635.41 |  |

Supplementary table 2. Approximate cost of reagents for tNGS TB drug prediction downstream processes based on reagents for the iSeq100 illumina sequencer.

| Work-step/category | Initial investment^1^ (USD) | |
| --- | --- | --- |
|  | with procurement in Germany | with procurement in South Africa |
| Plastics | 1,710.87 | 3,439.41 |
| Consumables for general use^2^ | 1,622.12 | 2,349.83 |
| DNA extraction | 3,026.66 | 3,522.92 |
| Targeted amplification | 12,840.00 | 28,663.37 |
| Library preparation | 6,905.78 | 10,567.86 |
| NGS | 7,511.40 | 9,659.10 |
| Shipment ^3^ | 6,420.00 | 1,070.00 |
| Total | 40,036.82 | 59,272.49 |

1 Including the initial procurement for a minimum of 200 samples. Some of the added items can be used for a much higher number of samples, but as there were no smaller volumes available on the market these had to be included in the initial investment. Costs per sample include only the main reagents for these steps and requires further validation, items for this implementation were procured in Germany and delivered with air freight, the costs in South Africa were only included for comparison. 2 items that can be used in one or more steps, which includes the following purposes: storing samples, buffers, chemicals, multiple steps, and controls. 3 Costs of shipment from Germany/South Africa to Namibia can vary, we based this value on our last shipments. For library preparation we used the Baym protocol [25], which is an adjusted version of Illumina’s NexteraXT library preparation kit.

Supplementary Table 3. Targeted NGS (Deeplex Myc-TB) cloud analysis output

| **Legends - based on the analysis output of Deeplex-Myc Cloud (https://deeplex.bluebee.com)** | | |
| --- | --- | --- |
|  |  |  |
| *“Sequencing result acceptability* | | |
| **+++** |  | All resistance-associated positions in database with enough data to identify mutations from 3-100% |
| **++** |  | All resistance-associated positions in database with enough data to identify mutations from 10-100% |
| **+** |  | All resistance-associated positions in database with enough data to identify mutations from 80-100% |
| **-** |  | One or more resistance-associated position in the database not covered - check target coverage (incl. for potential unreferred target deletion) |
| **ND** |  | Mycobacteria not detected |
| **NTM** |  | Nontuberculous mycobacteria detected |
|  |  |  |
| *Colours in resistotype* | | |
| **Grey** |  | Target with suboptimal/low coverage |
| **Red-yellow gradient** | | Drug resistance-associated variant or indel detected in target, with percent subpopulation according to colouring (red ≅ 100%, yellow ≅ 1%) |
| **Dark-light blue gradient** | | Non-synonymous uncharacterized variant or uncharacterized indel detected in target, with percent subpopulation according to colouring (dark blue ≅ 100%, light blue ≅ 1%) |
| **Green** |  | No resistance-associated and no uncharacterized variant or indel detected in target |
|  |  |  |
| *Antibiotics abbreviations* | | |
| **RIF** | Rifampicin |  |
| **INH** | Isoniazid |  |
| **PZA** | Pyrazinamide |  |
| **EMB** | Ethambutol |  |
| **SM** | Streptomycin |  |
| **FQ** | Fluoroquinolones | |
| **KAN** | Kanamycin |  |
| **AMI** | Amikacin |  |
| **CAP** | Capreomycin |  |
| **ETH** | Ethionamide |  |
| **LIN** | Linezolid |  |
| **BDQ** | Bedaquiline |  |
| **CFZ** | Clofazimine |  |
|  |  |  |
| Fluoroquinolones (FQ) include Levofloxacin (LEV), Ofloxacin (OFX), Moxifloxacin (MOX) and Ciprofloxacin (CIP)” | | |
